# Treatment Outcomes of New Tuberculosis Patients Hospitalized in Kampala, Uganda: A Prospective Cohort Study

**DOI:** 10.1371/journal.pone.0090614

**Published:** 2014-03-07

**Authors:** Bruce J. Kirenga, Jonathan Levin, Irene Ayakaka, William Worodria, Nancy Reilly, Francis Mumbowa, Helen Nabanjja, Grace Nyakoojo, Kevin Fennelly, Susan Nakubulwa, Moses Joloba, Alphonse Okwera, Kathleen D. Eisenach, Ruth McNerney, Alison M. Elliott, Roy D. Mugerwa, Peter G. Smith, Jerrold J. Ellner, Edward C. Jones-López

**Affiliations:** 1 Department of Medicine, Makerere University College of Health Sciences, Kampala, Uganda; 2 Medical Research Council–Uganda Virus Research Institute, Uganda Research Unit on AIDS, Entebbe, Uganda; 3 School of Public Health, University of the Witwatersrand, Johannesburg, Gauteng, South Africa; 4 Makerere University – Boston Medical Center Research Collaboration, Kampala, Uganda; 5 Department of Medicine, New Jersey Medical School – Rutgers University, Newark, New Jersey, United States of America; 6 Department of Microbiology, Makerere University College of Health Sciences, Kampala, Uganda; 7 Southeastern National Tuberculosis Center, Division of Mycobacteriology, Department of Medicine, University of Florida, Gainesville, Florida, United States of America; 8 Mulago Hospital Tuberculosis Clinic, Mulago Hospital, Kampala, Uganda; 9 Departments of Pathology and, Microbiology and Immunology, University of Arkansas for Medical Sciences, Little Rock, Arkansas, United States of America; 10 Department of Clinical Research, Faculty of Infectious and Tropical Diseases, London School of Hygiene & Tropical Medicine, London, England; 11 MRC Tropical Epidemiology Group, London School of Hygiene & Tropical Medicine, London, England; 12 Section of Infectious Diseases, Department of Medicine, Boston Medical Center and Boston University School of Medicine, Boston, Massachusetts, United States of America; Institute of Infectious Diseases and Molecular Medicine, South Africa

## Abstract

**Background:**

In most resource limited settings, new tuberculosis (TB) patients are usually treated as outpatients. We sought to investigate the reasons for hospitalisation and the predictors of poor treatment outcomes and mortality in a cohort of hospitalized new TB patients in Kampala, Uganda

**Methods and findings:**

Ninety-six new TB patients hospitalised between 2003 and 2006 were enrolled and followed for two years. Thirty two were HIV-uninfected and 64 were HIV-infected. Among the HIV-uninfected, the commonest reasons for hospitalization were low Karnofsky score (47%) and need for diagnostic evaluation (25%). HIV-infected patients were commonly hospitalized due to low Karnofsky score (72%), concurrent illness (16%) and diagnostic evaluation (14%). Eleven HIV uninfected patients died (mortality rate 19.7 per 100 person-years) while 41 deaths occurred among the HIV-infected patients (mortality rate 46.9 per 100 person years). In all patients an unsuccessful treatment outcome (treatment failure, death during the treatment period or an unknown outcome) was associated with duration of TB symptoms, with the odds of an unsuccessful outcome decreasing with increasing duration. Among HIV-infected patients, an unsuccessful treatment outcome was also associated with male sex (P = 0.004) and age (P = 0.034). Low Karnofsky score (aHR = 8.93, 95% CI 1.88 – 42.40, P = 0.001) was the only factor significantly associated with mortality among the HIV-uninfected. Mortality among the HIV-infected was associated with the composite variable of CD4 and ART use, with patients with baseline CD4 below 200 cells/µL who were not on ART at a greater risk of death than those who were on ART, and low Karnofsky score (aHR = 2.02, 95% CI 1.02 – 4.01, P = 0.045).

**Conclusion:**

Poor health status is a common cause of hospitalisation for new TB patients. Mortality in this study was very high and associated with advanced HIV Disease and no use of ART.

## Introduction

Improving tuberculosis (TB) treatment outcomes, including reducing mortality is part of the Millennium Development Goals. [1] Although TB-associated mortality was estimated to have decreased by 41% in 2011 from that reported in 1990, substantial morbidity and mortality is still recorded worldwide especially in low and middle income countries.[2] The number of TB deaths among HIV-uninfected people in 2011 was estimated as 990 000 (uncertainty interval of 0.84 million–1.1 million) [2]. An additional 430,000 deaths (uncertainty interval of 0.40 million–0.46 million) occurred among TB patients infected with HIV. [2]

Generally, TB is associated with higher morbidity and mortality among those infected with HIV. [3]–[5] Previous studies have attributed this to HIV/AIDS disease progression, [6] delayed diagnosis of TB and/or HIV, [7] drug-resistant TB (DR-TB), [5], [8] and a lack of immediate access to effective treatment. [9] A higher case fatality rate has also been associated with advanced age, extensive disease on chest X-ray and other co morbidities. [10]–[12] Several recommendations to improve outcomes of patients with TB have been implemented such as early diagnosis and directly observed therapy (DOT), [13] concurrent cotrimoxazole therapy [14], [15] and antiretroviral therapy (ART) in HIV infected patients have been implemented [16], [17]. However, whereas ART has been shown to be beneficial in TB/HIV infected patients, only about 30% of these patients access ART in most resource limited settings even after presentation to health care facilities with advanced HIV disease. [18]

The continued occurrence of high TB mortality rates despite effective treatment requires studies to investigate the determinants of mortality in different subsets of TB patients, particularly those at high risk of death. One such subset of patients is hospitalised new TB patients. The World Health Organization (WHO) defines new TB patients as individuals with no prior history of TB treatment or who have been treated for TB for less than one month. [19]–[21] Previous studies have documented excess mortality among hospitalized TB patients [22]–[24]. We have previously reported poor outcomes in a cohort of retreatment TB cases in which most of the mortality was attributable to drug-resistance [8]. In this study, we investigate the reasons for hospitalisation, poor treatment outcomes (treatment failure, relapse and mortality) and their predictors in a cohort of hospitalized new TB patients at a referral hospital in Kampala, Uganda.

## Methods

### Ethics

The study protocol was approved by the AIDS research sub-committee of the Uganda National Council of Science and Technology with additional clearance from the Institutional Review Boards of the University of Medicine and Dentistry of New Jersey and the London School of Hygiene & Tropical Medicine.

### Setting

The study was conducted at the 85-bed inpatient TB ward of the National TB and Leprosy Program (NTLP) Chemotherapy Centre at Mulago National Referral Hospital in Kampala, Uganda. The NTLP Centre serves both as a local treatment clinic (largest in Kampala) and as the national referral centre (approximately one third of attendees are referral cases). This unit has a dedicated ward for admission of TB patients for retreatment (daily injection of streptomycin), for management of complications of TB and for new TB patients with severe disease who need hospitalization.

### Patients

All new TB patients hospitalized from July 2003 to January 2007 were eligible for inclusion in the study, provided they were 18 years or older and gave written informed consent. Eligible subjects were included in the study if they provided sputum specimens that were positive for acid-fast bacilli (AFB) on smear microscopy and TB was confirmed subsequently by growth of *M*. *tuberculosis* in culture. A standard questionnaire was administered to patients regarding risk factors for TB disease, and a presence or absence of symptoms and their duration, clinical examination was performed on all patients at enrolment and a postero-anterior chest radiograph was graded by an experienced clinician for extent of disease on a four-category ordinal scale. HIV testing was performed at study enrolment after counselling and informed consent. Patients that were found to be HIV seropositive had CD4 counts performed.

### Treatment and Follow up

All patients were offered TB treatment based on the standard 8-month WHO-recommended Category I treatment regimen comprising 2 months of rifampicin (R), isoniazid (H), pyrazinamide (Z), ethambutol (E), followed by 6 months of H and E (2 RHZPE/6 HE) [21]. In accordance with existing national guidelines, HIV infected patients received cotrimoxazole prophylaxis and those with a CD4+ cell count less than 200 cells/µl were referred for ART though access to ART was limited in Kampala before 2005. Patients received daily DOT while admitted to the hospital and were discharged from the hospital at the discretion of the attending physician when judged to be sufficiently recovered to continue treatment on an ambulatory basis. After hospital discharge, subjects were seen monthly during their 8-month TB treatment course, at month 9, and quarterly thereafter for the duration of the study. Patients who did not keep their scheduled visits were traced at their homes. During follow-up visits, home health visitors assessed treatment adherence through treatment card review, monthly pill counts, and patient self-reporting. Adherence was categorized into a five-level ordinal scale (fully adherent, missed less than 20% of doses, missed about one half of the doses, missed most doses, and not adherent at all). The worst assessment at any visit was taken as the summary measure of adherence for each subject. Because of small numbers of patients in some categories, patients were classified in the analyses as either “mostly adherent” (consisting of the first two categories) or “missed half or more” (consisting of the remaining three categories).

### Laboratory Methods

Sputum specimens were processed with the standard digestion and decontamination method using NALC/Na citrate/NaOH. Centrifuged pellets were re-suspended in a phosphate buffer solution and used to prepare smears and cultures on 7H10 Middlebrook agar and liquid media. Sputum smear microscopy was performed using auramine O fluorescent stain and reported according to the United States Centers for Disease Control and Prevention (CDC) microscopy grading scheme (negative or 1+ to 4+). [19] Sputum sediments were cultured in either BACTEC™ 460 or BACTEC MGIT™ 960 (Becton Dickinson Diagnostic Instrument Systems, Sparks, USA) according to the manufacturer's recommendations [25], [26]. Confirmation of *M. tuberculosis* complex was determined by either the BACTEC NAP test (BD, Sparks, USA) or PCR for IS*6110* as previously described. [27] The number of days from inoculation of 12B media until positive (growth index >30) BACTEC culture was determined for baseline specimens. Initial *M. tuberculosis* isolates obtained from each patient at the time of recruitment were subjected to drug susceptibility testing (DST) for S, R, H, E and Z using BACTEC™ 460 or MGIT 960. For quality assurance of DST, 245 specimens were also tested at the CDC with over 95% concordance. The radiometric BACTEC™ 460 was used through July 2006 when it was replaced by the fluorometric MGIT™ 960. During follow-up, two sputum samples were collected for AFB smear microscopy and culture at each of five pre-defined time points (months 1, 2, 5, 8 and 12); additional samples were collected at later visits if patients had a productive cough.

### Treatment outcomes

Following WHO treatment outcome definitions, [28] subjects were classified as *cured* if they completed treatment and had a negative culture on solid medium at the end of treatment. Subjects were classified as having *completed* treatment if they finished the full 8 months of therapy and were found to be free of TB symptoms at their first post-treatment follow-up visit, but had no culture results at the end of treatment. They were classified as having an *unknown* outcome if they were lost to follow-up (the majority of these subjects were treatment defaulters) and, classified as *treatment failures* if they were culture positive at month 8 or, if they were culture positive at month 5 with no culture at month 8 and we had no confirmation that they were free of TB after the end of treatment. At 8 months, a *successful* TB treatment outcome was defined as cure or treatment completion with no evidence of remaining disease, and *unsuccessful* outcome as treatment failure, death during treatment, or unknown outcome. During follow up, subjects were evaluated for TB recurrence and vital status was assessed at the end of scheduled follow-up, which was 24 months after the initiation of TB treatment, or the close of the study.

### Statistical Analysis

The objectives of the statistical analyses were to describe the treatment outcomes and mortality of HIV-uninfected and HIV-infected subjects and to find factors associated with treatment outcome and mortality. Factors were considered for inclusion in statistical models as risk factors or confounders based on prior knowledge or biological plausibility. [29] The number of potential explanatory variables included in the analyses was restricted because of the relatively small study size. A backward elimination approach was used among the candidate regressors, bearing in mind the need to restrict the number of regressors so that the number of parameters in the final model was, at most, about 10% of the number of either subjects with unsuccessful treatment outcomes or subjects who died. For finding factors associated with an unsuccessful treatment outcome (treatment failure, death during the treatment period or an unknown outcome), multiple logistic regression models were fitted both to all patients and separately for HIV-infected patients. Survival analysis methods were used to investigate factors associated with mortality. Kaplan- Meier plots were drawn to compare mortality rates between HIV-infected and HIV-uninfected subjects and, in the case of HIV-infected subjects, to examine the impact of baseline CD4 count and ART on mortality. Cox proportional hazards regression models were fitted to find factors associated with mortality. Separate models were fitted for HIV-uninfected and HIV-infected subjects since the composite variable for baseline CD4 count and ART status was not applicable for HIV-uninfected subjects. This composite variable with five levels, namely “CD4 ≥ 200”, “CD4 50 – 199 no ART”, “CD4 50 – 199 ART”, “CD4<50 no ART” and “CD4<50 ART”, was a time varying covariate as some subjects started ART sometime after commencing anti-tuberculosis chemotherapy. The composite variable was used rather than separate variables for baseline CD4 count and ART as no subject with a baseline CD4 count of 200 cells/µL or above started ART. The proportional hazards assumption was checked using graphical methods as well as by the test of Grambsch and Therneu.[30] All analyses were carried out using Stata release 11.2 (College Station, TX: StataCorp LP).

## Results

### Study patients

We enrolled 96 hospitalized TB patients of whom 64 (66.7%) were HIV -infected. Table 1 compares characteristics of participants at the time of enrolment by HIV status. The mean age of HIV-uninfected was 32.3 years, which was similar to that of HIV-infected (33.1 years). There was a higher proportion of females among HIV-infected than among the HIV-uninfected (61% *vs.* 22%). The duration of symptoms before hospitalization was shorter among HIV-infected (median 12 weeks, IQR 8-16) than among HIV-uninfected (median 16 weeks, IQR 8 – 24). The prevalence of different symptoms at the time of time of admission was similar in the 2 groups. Cavitation on chest X-ray was observed more frequently in HIV-uninfected (87%) compared to HIV-infected (20%) and a higher proportion of HIV-uninfected were judged to have severe disease on the chest X-ray (90% *vs.* 43%). HIV-infected patients generally presented with advanced AIDS (median CD4 cell count 58 cells/µl). Six patients (2 HIV-uninfected and 4 HIV-infected) had mono-resistance to INH or RIF and one patient (HIV uninfected) had primary MDR-TB.

**Table 1:** Baseline Characteristics of 96 Hospitalized New TB Patients by HIV status in Kampala, Uganda.

Factor	Level	HIV uninfected (n = 32)	HIV infected (n = 64)
		– n (%) (unless specified) –	
Age (years)	Mean (SD)	32.3 (13.8)	33.1 (8.2)
Sex	Female	7 (22%)	39 (61%)
	Male	25 (78%)	25 (39%)
BMI (kg/m2)	Mean (SD)	16.6 (3.1)	17.5 (3.5)
Karnofsky score	70+	20 (62.5%)	36 (56%)
	≤60	12 (37.5%)	28 (44%)
Duration of TB symptoms (weeks)	Median (IQR)	16 (8 – 24)	12 (8 – 16)
Drug resistance status at baseline	Sensitive to INH and RIF	29 (91%)	60 (94%)
	Resistance to INH or RIF	2 (6%)	4 (6%)
	MDR	1 (3%)	0
Extent of disease on chest radiograph	Normal	0	7 (11%)
	Minimal	0	14 (22%)
	Moderate	3 (10%)	15 (24%)
	Severe	28 (90%)	27 (43%)
Cavitation	Absent	4 (13%)	49 (80%)
	Present	27 (87%)	12 (20%)
Miliary infiltrate	Absent	30 (97%)	58 (92%)
	Present	1 (3%)	5 (8%)
Sputum AFB smear microscopy grade	1+	3 (9%)	4 (6%)
	2+	3 (9%)	14 (22%)
	3+	5 (16%)	11 (17%)
	4+	21 (66%)	35 (55%)
BACTEC days-to-positive culture	≥7 days	14 (44%)	32 (50%)
	<7 days	18 (56%)	32 (50%)
CD4 cell count (cells/µL)	Median (IQR)	-	58 (18 – 106)
Symptoms (multiple responses allowed)	Fever	20 (62%)	51 (80%)
	Rigors	9 (28%)	7 (11%)
	Night sweats	21 (66%)	46 (72%)
	Cough	32 (100%)	63 (98%)
	Produce sputum	32 (100%)	62 (97%)
	Purulent sputum	18 (56%)	29 (45%)
	Hemoptysis	5 (16%)	2 (3%)
	Dyspnea	21 (66%)	52 (81%)
	Chest Pain	22 (69%)	46 (72%)
	Loss of appetite	21 (66%)	53 (83%)
	Diarrhoea	2 (6%)	3 (5%)
	Weight Loss	26 (81%)	60 (94%)
	Swollen Glands	0	12 (19%)
	Malaise	23 (72%)	52 (81%)
Reason for hospitalization	Low Karnofsky score	15 (47%)	46 (72%)
	Diagnostic evaluation	8 (25%)	9 (14%)
	Concurrent Illness	2 (6%)	10 (16%)
	Haemoptysis	3 (9%)	0
	Social support	2 (6%)	0
	Drug side effects	0	0
	Two reasons for admission	3 (9%)	2 (3%)
	Other reasons	5 (16%)	1 (2%)

Patients were hospitalized for a median period of 32 days (IQR 22 – 56). The most frequent reason for hospitalisation was low Karnofsky score, with this being more common among HIV-infected patients (72% vs. 47% for HIV-uninfected patients). The proportion of patients admitted for diagnostic evaluation was higher among the HIV-uninfected (25% vs. 14%). Ten (16%) HIV-infected patients were admitted due to concurrent illness and three HIV-uninfected patients were admitted for haemoptysis and two for social reasons (Table 1).

### Follow up and treatment adherence

**Figure 1:**
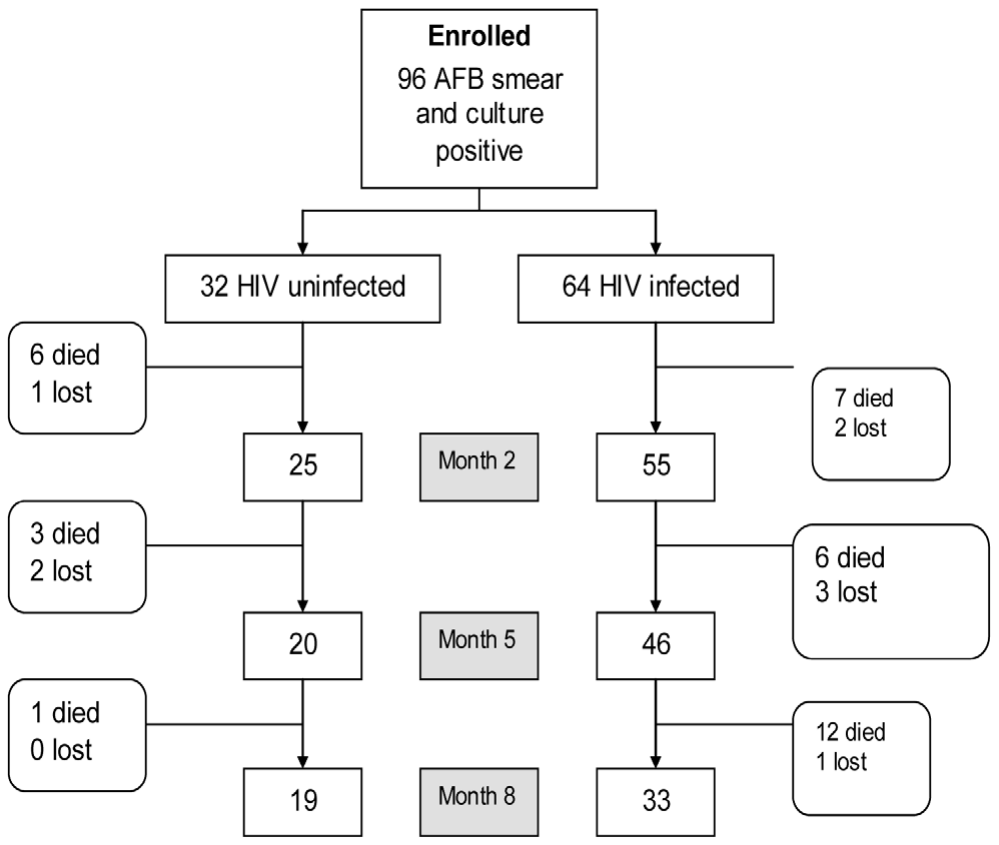
Study profile during the 8-month TB treatment period. Note: Of the 19 HIV uninfected who were still in the study at month 8, 17 were classified as cured/completed, 1 failed treatment and was put on a retreatment regimen and 1 had an unknown outcome. Of the 33 HIV infected who were still in the study at month 8, 22 were classified as cured or completed treatment, 7 failed treatment (of whom 5 were put on retreatment) and 4 had unknown outcomes.

The follow-up of participants over the course of their 8-month anti-tuberculosis therapy is shown in Figure 1. During TB treatment, 9 (9%) participants were lost to follow-up and 35 (36%) died. After completing treatment, the 52 (54%) patients retained in the treatment cohort were followed for between 1 and 43 months (median 21 months, IQR 5 – 37). A further 17 (18%) participants died (16 HIV-infected, one HIV uninfected) and two (3.4%) were lost to follow-up (one HIV-infected and one HIV uninfected). The remaining patients either completed a total of 24 months of follow-up or were seen at a follow-up visit during the last three months of the study. Adherence to TB treatment during the continuation phase was similar for HIV-uninfected and HIV-infected patients. Adherence information was missing in 12 HIV-uninfected and 16 HIV-infected patients, mostly because the patients had died or were lost to follow-up before adherence information could be collected.

### Tuberculosis Treatment Outcomes

TB treatment outcomes by HIV status and potential explanatory factors are shown in Table 2. The results of fitting multiple logistic regression models, firstly to all patients and secondly to the 64 HIV-infected patients as a planned sub-group analysis, are shown in Table 3. Overall 39 (41%) patients had a successful treatment outcome (cured or completed treatment), with the proportion being higher for the HIV uninfected (53%) than for the HIV infected (34%). Factors associated with a favourable outcome among HIV-uninfected patients were age (with patients aged 30–40 years being less likely to have a favourable outcome) and sputum bacterial load as measured by MGIT DTP, with those patients for whom the DTP was 7 days or more being more likely to have a favourable outcome. Among the HIV infected patients there was a similar age effect, with those patients aged 31–40 being less likely to have a favourable outcome and an effect of sex, with female patients being more likely to have a favourable outcome. In the multiple logistic regression models for an unfavourable outcome, the only factor that reached statistical significance in the overall model was the duration of TB symptoms (Table 3), with a longer interval being associated with more favourabe outcomes with an adjusted Odds Ratio (aOR) of 0.93 for a one week increase in the duration of symptoms (95% c.i. 0.89 – 0.98; P = 0.002). The odds of having an unfavourable outcome were higher for the HIV infected patients although this did not approach statistical significance (aOR = 1.70; 95% c.i. 0.68 – 4.29; P = 0.26). In the sub-analysis restricted to the HIV co-infected patients, both age and sex were associated with a poor outcome, with those aged 30–40 years being most likely to have an unfavourable outcome, and males more likely than females to have an unfavourable outcome (aOR = 7.77; 95% c.i. 1.55 – 38.8; P = 0.004).

**Table 2:** TB outcomes by HIV status and other factors.

Study Group		Treatment Outcome – n (%) –				
		Cured/Completed	Died before Month 8	Failed	Unknown	Totals
HIV-uninfected	(n = 32)	17 (53%)	10 (31%)	1(3%)	4 (13%)	32
Age (years)	≤30	11 (55%)	5 (25%)	1 (5%)	3 (15%)	20
	>30–40	2 (40%)	2 (40%)	0	1 (20%)	5
	>40	4 (57%)	3 (43%)	0	0	7
Sex	Female	3 (43%)	2 (29%)	1 (14%)	1 (14%)	7
	Male	14 (56%)	8 (32%)	0	3 (12%)	25
Resistance status	Sensitive to RH	15 (52%)	9 (31%)	1(3%)	4 (14%)	29
	Resistant to R or H or both	2 (67%)	1 (33%)	0	0	3
Adherence in continuation phase *	(Almost/)Fully compliant	11 (73%)	1 (7%)	1 (7%)	2 (13%)	15
	Missed half or more	4 (80%)	0	0	1 (20%)	5
Duration of TB symptoms (months)	<3 months	4 (36%)	2 (18%)	1(9%)	4 (36%)	11
	3–6 months	6 (43%)	8 (57%)	0	0	14
	> 6 months	7 (100%)	0	0	0	7
BMI kg/m2 (grouped)	≤15	5 (62.5%)	2 (25%)	0	1 (12%)	8
	>15–18.5	6 (50%)	3 (25%)	0	3 (25%)	12
	>18.5	6 (50%)	5 (42%)	1(8%)	0	12
Extent of disease on X-ray *	Normal	0	0	0	0	0
	Minimal	0	0	0	0	0
	Moderate	1 (33%)	1 (33%)	0	1 (33%)	3
	Severe	15 (54%)	9 (32%)	1 (4%)	3 (11%)	28
Cavitation *	No	1 (25%)	2 (50%)	0	1 (25%)	4
	Yes	15 (56%)	8 (30%)	1 (4%)	3 (11%)	27
Days to positivity	7+	9 (64%)	2 (14%)	1 (7%)	2 (14%)	14
	<7	8 (44%)	8 (44%)	0	2 (11%)	18
HIV infected	(n = 64)	22 (34%)	25 (39%)	7 (11%)	10 (16%)	64
Age (years)	≤30	12 (43%)	9 (32%)	3 (11%)	4 (14%)	28
	>30–40	5 (19%)	13 (50%)	3 (12%)	5 (19%)	26
	>40	5 (50%)	3 (30%)	1 (10%)	1 (10%)	10
Sex	Female	18 (46%)	16 (41%)	2 (5%)	3 (8%)	39
	Male	4 (16%)	9 (36%)	5 (20%)	7 (28%)	25
Resistance status	Sensitive to RH	20 (33%)	23 (38%)	7 (12%)	10 (17%)	60
	Resistant to R or H or both	2 (50%)	2 (50%)	0	0	4
Adherence in continuation phase *	(Almost/)Fully compliant	16 (44%)	10 (28%)	5 (14%)	5 (14%)	36
	Missed half or more	5 (42%)	4 (33%)	0	3 (25%)	12
Duration of TB symptoms (months)	<3 months	12 (30%)	16 (40%)	4 (10%)	8 (20%)	40
	3–6 months	7 (35%)	9 (45%)	2 (10%)	2 (10%)	20
	> 6 months	3 (75%)	0	1 (25%)	0	4
BMI kg/m2 (grouped)	≤15	7 (41%)	7 (41%)	2 (12%)	1 (6%)	17
	15–18.5	12 (35%)	13 (38%)	4 (12%)	5 (15%)	34
	>18.5+	2 (17%)	5 (42%)	1 (8%)	4 (33%)	12
Extent of disease on X-ray *	Normal	2 (29%)	3 (43%)	1 (14%)	1 (14%)	7
	Minimal	4 (29%)	7 (50%)	2 (14%)	1 (7%)	14
	Moderate	6 (40%)	7 (47%)	0	2 (13%)	15
	Severe	10 (37%)	8 (30%)	4 (15%)	5 (19%)	27
Cavitation *	No	18 (37%)	20 (41%)	7 (14%)	4 (8%)	49
	Yes	2 (17%)	5 (42%)	0	5 (42%)	12
Days to positivity	7+	10 (31%)	14 (44%)	3 (9%)	5 (16%)	32
	<7	12 (38%)	11 (34%)	4 (12%)	5 (16%)	32

**Table 3:** Results of fitting multiple logistic regression models for factors associated with unsuccessful outcomes.

(1) Overall model				
Factor	Level	Adjusted Odds Ratio^1^	95% confidence limits	P-value
HIV status	Uninfected	1	Reference level	0.26
	Infected	1.7	( 0.68; 4.29)	
Duration of TB symptoms	1 week increase	0.93	(0.89; 0.98)	0.002
(2) Model for HIV-infected patients only				
Age group	18 – 30	1	Reference level	0.034
	>30 – 40	2.68	0.73; 9.83	
	> 40	0.25	0.04; 1.69	
Sex	Female	1	Reference level	0.004
	Male	7.77	( 1.55; 38.8)	

1 Odds ratios adjusted for other terms in the model

### Survival

**Figure 2:**
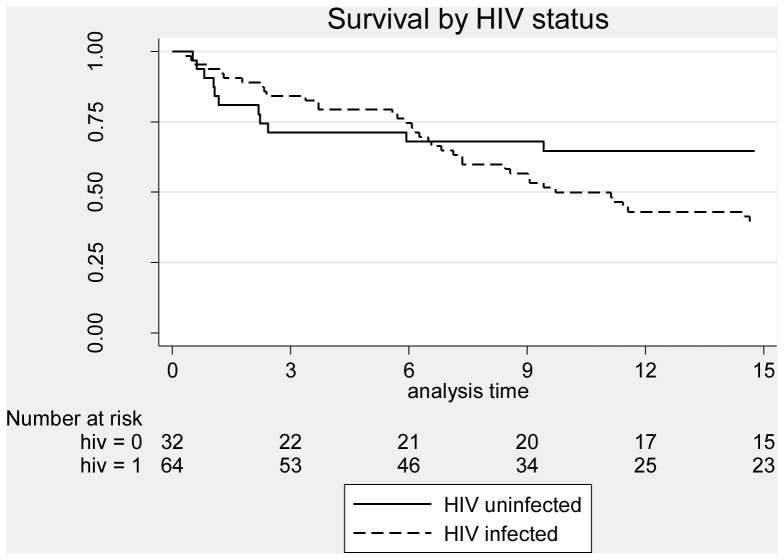
Survival of subjects by HIV status.

**Figure 3:**
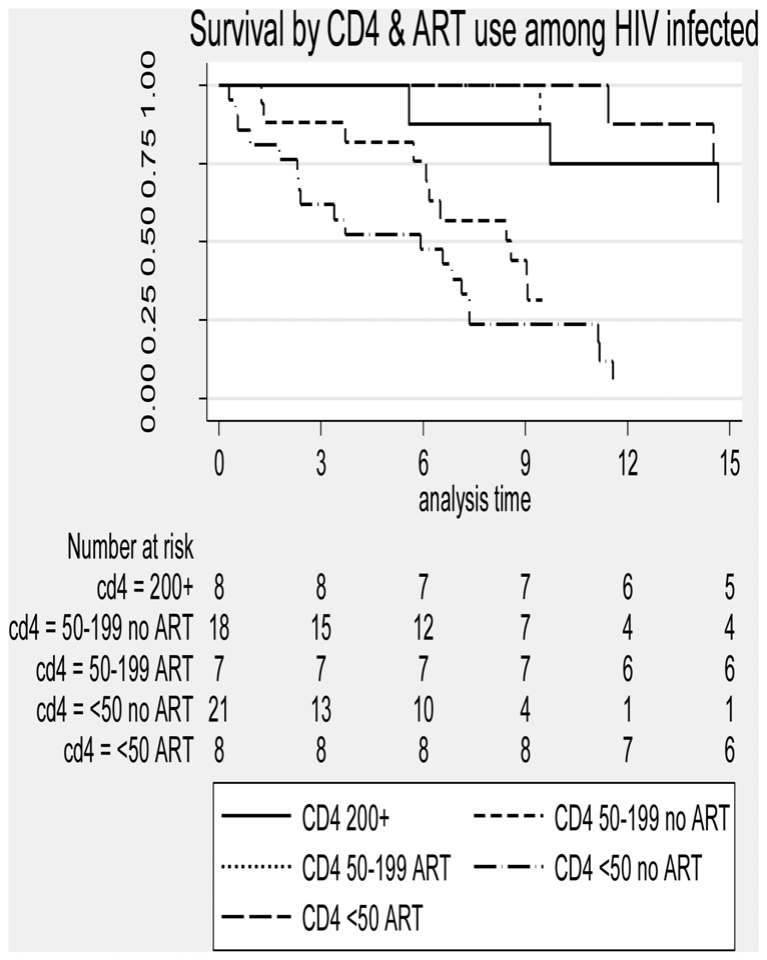
Mortality by CD4 cell count at baseline and ART use among HIV-infected subjects.

Mortality rates by HIV status and potential explanatory factors are shown in table 4. Overall, 52 (54%) patients died of which 35 (36%) died during TB treatment and 17 (18%) died during the post TB treatment period. The mortality rate (per 100 person years at risk) was significantly higher for the HIV infected patients (41 deaths, rate 46.9 per 100 pyar) than for the HIV-uninfected patients (11 deaths, rate 19.7 per 100 pyar) (Figure 2). Among HIV-uninfected patients, the mortality rate was higher for those aged 30–40 years (43.0 per 100 pyar), for those with a Karnofsky score on admission of 60 or less (72.6 per 100 pyar), those without cavitation on chest X-ray (38.7 per 100 pyar) and for those who had TB symptoms for 3–6 months. Among HIV-infected patients, the rate was higher for those aged 30–40 (70.0 per 100 pyar), for males (50.7 per 100 pyar), for those with a BMI above 18.5 kg/m^2^, for those with a Karnofsky score on admission of 60 or less (94.6 per 100 pyar), for those with some anti-TB drug resistance (91.2 per 100 pyar) and for those with an AFB sputum smear grade of 3+ (72.0 per 100 pyar). Mortality rates were also significantly higher for patients with CD4 cell counts below 200 cells/µL who were not on ART (rate 76.5 per 100 pyar) for those with CD4 counts 50–199 (rate 194 per 100 pyar) and for those with CD4 counts below 50 (Figure 3). In the multivariable Cox regression model for factors affecting survival among HIV-uninfected patients (table 5), the only factors associated with increased risk of death were baseline Karnofsky score, with mortality higher for those with a Karnofsky score of 60 or less at baseline [adjusted Hazard Ratio (aHR) 8.93, 95% c.i. 1.88 – 42.4; P = 0.001], and DTP which was marginally significant, with subjects with DTP<7 days having higher mortality (aHR = 3.44; 95% c.i. 0.73 – 16.1; P = 0.08). Among HIV-infected patients, the factors independently associated with mortality were Karnofsky score, with patients with a Karnofsky score of 60 or less having a higher mortality rate (aHR = 2.02; 95% c.i. 1.02 – 4.01; P = 0.045), and the composite factor of CD4 count and ART, with overwhelming evidence (P<0.001) that mortality varied with the level of this factor, with those with CD4 counts below 200 who were not on ART at increased risk compared to patients with a CD4 above 200 (aHR = 3.59 for those with CD4 50–199 and aHR = 6.90 for those with a CD4<50) and those on ART at a reduced risk even compared to those with a CD4 above 200 (who were not on ART due to the prevailing treatment guidelines at the time of the study).

**Table 4:** Mortality by HIV status and potential explanatory factors.

				HIV-uninfected			HIV infected
Factor	Level	n	Deaths	Mortality rate per 100 pyo (95% c.i.)	n	deaths	Mortality rate per 100 pyo (95% c.i.)
Overall		32	11	19.7 (10.9; 35.5)	64	41	46.9 (34.6; 63.7)
Age	<30	20	5	13.2 (5.5; 31.9)	28	17	40.1 (24.9; 64.5)
(years)	30–40	5	3	43.0 (13.9; 133)	26	19	70.0 (44.7; 109)
	>40	7	3	26.7 (8.6; 82.7)	10	5	28.1 (11.7; 67.4)
Sex	Female	7	2	13.0 (3.2; 51.9)	39	25	44.8 (30.3; 66.3)
	Male	25	9	22.2 (11.6; 42.7)	25	16	50.7 (31.1; 82.8)
BMI1 (kg/m2)	≤15	8	2	15.2 (3.8; 61.0)	17	10	33.9 (18.2; 63.0)
	>15–18.5	12	4	19.3 (7.2; 51.4)	34	20	41.7 (26.9; 64.7)
	>18.5	12	5	22.7 (9.4; 54.5)	12	10	111 (59.8; 206)
Karnofsky Score	70 +	20	2	4.6 (1.1; 18.4)	36	16	26.3 (16.1; 42.9)
	≤ 60	12	9	72.6 (37.8; 139)	28	25	94.6 (63.9; 140)
Resistance Status	Sens HR	29	10	19.6 (10.6; 36.5)	60	38	45.2 (32.9; 62.1)
	Res H/R or both	3	1	20.0 (2.8; 142)	4	3	91.2 (29.4; 282)
Sputum smear grade	1+ or 2+	6	1	10.8 (1.5; 76.3)	18	11	41.2 (22.8; 74.3)
	3+	5	1	8.0 (1.1; 56.6)	11	9	72.0 (37.5; 138)
	4+	21	9	26.4 (13.7; 50.8)	35	21	43.6 (28.5; 66.9)
Extent of disease on chest X-ray	Norm/Min	0	0	-	21	14	64.6 (38.3; 109)
	Moderate	3	1	19.4 (2.7; 138)	15	9	36.1 (18.8; 69.4)
	Severe	28	10	21.2 (11.4; 39.5)	27	17	43.8 (27.2; 70.4)
Cavitation	No	4	2	38.7 (9.7; 154)	49	31	43.9 (30.9; 62.4)
	Yes	27	9	19.1 (10.0; 36.8)	12	8	66.7 (33.3; 133)
Duration of TB symptoms	<3 months	11	3	14.7 (4.7; 45.5)	40	27	49.9 (34.2; 72.8)
	3–6 months	14	8	44.1 (22.1; 88.2)	20	12	48.7 (27.7; 85.7)
	> 6 months	7	0	0	4	2	23.3 (5.8; 93.1)
DTP	≥7 days	14	2	5.9 (1.5; 23.6)	32	19	40.7 (26.0; 63.8)
	< 7 days	18	9	40.9 (21.3; 78.6)	32	22	54.1 (35.6; 82.1)
ART & CD4 group	CD4≥200				8*	3	17.0 (5.5; 52.7)
	CD4 50–199, no Art				18	12	76.5 (43.4; 130)
	CD4 50–199, ART				7	1	4.9 (0.68; 34.5)
	CD4<50, no ART				21	20	194 (120; 300)
	CD4<50. ART				8	3	15.1 (4.9; 46.7)
Adherence	Almost/fully	15	2	6.4 (1.6; 25.4)	36	23	40.0 (26.6; 60.2)
	Missed half	5	0	0	12	6	29.9 (13.4; 66.6)
	No Info	12	9	114 (59.2; 218)	16	12	122 (69.1; 214)

* This is a time varying covariate; numbers are those for whom the category was their final category

**Table 5:** Mortality – results of fitting Cox proportional hazards regression models by HIV status.

Factor	Level	Hazard Ratio (95% c.i.)	P-value
(1) HIV-uninfected subjects (n = 32)			
Karnofsky score	70+	1	0.001
	≤ 60	8.93 (1.88; 42.4)	
DTP	≥ 7	1	0.08
	< 7 days	3.44 (0.73; 16.1)	
(2) HIV-infected subjects (n = 64)			
Baseline CD4 & ART	CD4 ≥ 200 cells/mm3	1	<0.001
	CD4 50–199, no ART	3.59 (0.97; 13.2)	
	CD4 50–199, ART	0.28 (0.029; 2.73)	
	CD4<50, no ART	6.90 (1.89; 25.2)	
	CD4<50, ART	0.77 (0.15; 3.85)	
Karnofsky Score	70 +	1	0.045
	≤ 60	2.02 (1.02; 4.01)	

## Discussion

In this prospective cohort study in Uganda we documented that most new TB patients are hospitalized because of severe TB illness, for diagnostic evaluation or concurrent illness. Unsuccessful treatment outcome and mortality were high: 59% and 54.2% respectively. The major determinants of mortality were severe disease at the time of hospitalization, HIV infection and no ART in those with CD4<200 cells/µl, especially in those with CD4<50 cells/µl.

High mortality among TB patients in general and hospitalized patients in particular is consistently reported in many studies [22]–[24], [31]–[34]. It is higher in resource limited settings and among those that are HIV-infected. Some of our findings are similar to those reported in the above studies but there are some important differences. Firstly, the observed mortality is much higher even when compared to other African countries. For example in Nigeria mortality was 18% [22]. This could be attributed to the high HIV prevalence in our cohort (over 50%). Most of these studies were retrospective in nature while ours was prospective with a long follow up of two years. A systematic review published in 2011 on this subject concluded that prospective studies are needed as most of the available studies were retrospective in nature and relied on registries and charts [35]. This study also shows that much of the mortality among HIV-uninfected TB patients occurred earlier during TB treatment, while mortality in HIV- infected patients was evenly spread through the follow up period. Early mortality could be due to TB disease diminishing with longer duration on TB treatment. The reduction could be due to response to TB treatment since the prevalence of drug resistance was low in this cohort. Persistent mortality among HIV-infected TB patients can be explained by HIV disease progression, as access to ART in the country was limited at the time of the study; indeed no use of ART was found to be strongly associated with mortality in this cohort. [36]

As regards to risk factors for death, our findings are similar to previously reported TB mortality studies where risk factors differ by setting [22]–[24], [31]–[34]. In developed settings, non-communicable diseases comorbidities, alcohol and drug abuse, and homelessness are the major factors; in less developed settings, the major factors are HIV and delayed presentation for care [22]–[24], [31]–[34], [37], [38]. Although HIV is a major driver of mortality in TB, mortality was also high among HIV-uninfected patients in this study (34.5% of the HIV-uninfected cohort died). A similar result was found in Philippines where 30% of HIV-uninfected patients with TB died in hospital, most during the few days of hospitalization [39]. Therefore drivers of TB mortality other than HIV need to be addressed if TB related mortality, a key indicator recognized by the MDG, is to be reduced [1]. Factors such as delayed diagnosis are a reflection of poor health seeking behaviour and inaccessible health services should be investigated and addressed. Delayed diagnosis of TB and/or HIV has also been previously described as a predictor of mortality among TB patients [7], [40], [41]. The consistent finding of delayed diagnosis as a predictor of mortality in previous studies highlights the need for improved case detection and expansion of TB diagnostic and treatment services near to the populations most at risk if TB related mortality is to be reduced. Lack of immediate access to effective treatment has been described as a major reason for increased mortality in TB/HIV patients [9]. However the finding of a reduced risk of death with increasing duration of symptoms in the present study is rather paradoxical and needs further investigation in larger prospective cohort studies. The poor treatment outcomes were principally dictated by the high mortality rates since more than one third of the patients (36%) died during the period of hospitalization. There were however, low rates of drug-resistance and this was not a significant contributor to the mortality in this cohort.

Results from this study indicate that failure to start ART earlier on during TB treatment is associated with increased mortality among patients with CD4<200 cells/µL; mortality rates were highest among patients with CD4<50 cells/µL who are not on ART. This finding is similar to that from recently concluded clinical trials in which delayed ART was associated with much higher mortality among patients with CD4<50 cells/µL [42]–[44]. For patients with advanced immune suppression the possibility of initiating ART during hospitalization for TB treatment should be considered although the possibility of drug toxicity and immune reconstitution should be weighed against the benefits of treatment as these have been reported in studies investigating TB/HIV co-treatment[43], [45].

This study had some limitations. The study participants were a highly selected population, as most newly diagnosed TB patients are not admitted to hospital, and the findings cannot therefore be extrapolated to other TB patients in ambulatory settings. Also the study size was small with respect to HIV-uninfected patients and an exhaustive investigation of known predictors of poor treatment outcomes such as hypoalbunaemia, hyponatraemia, anaemia, neutrophilia and lymphopenia were not investigated. It must also be noted that the study started before the availability of ART in the public sector in Uganda and that under current guidelines all HIV co-infected patients would receive ART especially so if their CD4 cell counts are below 200 cells/µL.

In conclusion, this study has demonstrated that poor health status is a major cause of hospitalisation for new TB patients. Once hospitalised, these patients have a high frequency of poor treatment outcome and mortality that appears to be driven by severity of illness, HIV/AIDS and no use of antiretroviral therapy if CD4 cell count is below 200 cells/µl. Early case detection and effective treatment for both tuberculosis and HIV would probably lead to reduction of hospitalization and reduce TB related mortality.

## References

[1] Steingart KR, Sohn H, Schiller I, Kloda LA, Boehme CC, et al (2013) Xpert(R) MTB/RIF assay for pulmonary tuberculosis and rifampicin resistance in adults. Cochrane Database Syst Rev 1: CD009593. 10.1002/14651858.CD009593.pub2 PMC4470352 23440842

[2] Mosimaneotsile B, Talbot EA, Moeti TL, Hone NM, Moalosi G, et al (2003) Value of chest radiography in a tuberculosis prevention programme for HIV-infected people, Botswana. Lancet 362: 1551–1552. 14615113 10.1016/s0140-6736(03)14745-9

[3] Corbett EL, Marston B, Churchyard GJ, De Cock KM (2006) Tuberculosis in sub-Saharan Africa: opportunities, challenges, and change in the era of antiretroviral treatment. Lancet 367: 926–937. 16546541 10.1016/S0140-6736(06)68383-9

[4] Lawn SD, Wood R (2006) The epidemic of HIV-associated tuberculosis in sub-Saharan Africa: does this also impact non-HIV-infected individuals? Aids 20: 1787–1788. 16931949 10.1097/01.aids.0000242831.27990.da

[5] Gandhi NR, Shah NS, Andrews JR, Vella V, Moll AP, et al HIV coinfection in multidrug- and extensively drug-resistant tuberculosis results in high early mortality. Am J Respir Crit Care Med 181: 80–86. 19833824 10.1164/rccm.200907-0989OC

[6] Whalen CC, Nsubuga P, Okwera A, Johnson JL, Hom DL, et al (2000) Impact of pulmonary tuberculosis on survival of HIV-infected adults: a prospective epidemiologic study in Uganda. Aids 14: 1219–1228. 10894287 10.1097/00002030-200006160-00020 PMC2869086

[7] Palmieri F, Girardi E, Pellicelli AM, Rianda A, Bordi E, et al (2002) Pulmonary tuberculosis in HIV-infected patients presenting with normal chest radiograph and negative sputum smear. Infection 30: 68–74. 12018472 10.1007/s15010-002-2062-9

[8] Jones-Lopez EC, Ayakaka I, Levin J, Reilly N, Mumbowa F, et al (2011) Effectiveness of the standard WHO recommended retreatment regimen (category II) for tuberculosis in Kampala, Uganda: a prospective cohort study. PLoS Med 8: 15. 10.1371/journal.pmed.1000427 PMC3058098 21423586

[9] Yen YF, Yen MY, Shih HC, Deng CY (2012) Risk factors for unfavorable outcome of pulmonary tuberculosis in adults in Taipei, Taiwan. Trans R Soc Trop Med Hyg 106: 303–308. 22387265 10.1016/j.trstmh.2012.01.011

[10] Naidoo S, Douglas D (2008) Admission trends of adult TB patients at one of the largest tuberculosis hospitals in South Africa: from 2001 to 2003. Southern African Journal of Epidemiology and Infection 22: 8–12.

[11] Abos-Hernandez R, Olle-Goig JE (2002) Patients hospitalised in Bolivia with pulmonary tuberculosis: risk factors for dying. Int J Tuberc Lung Dis 6: 470–474. 12068977 10.5588/09640569512959

[12] Duarte EC, Bierrenbach AL, Barbosa da Silva J Jr, Tauil PL, de Fatima Duarte E (2009) Factors associated with deaths among pulmonary tuberculosis patients: a case-control study with secondary data. J Epidemiol Community Health 63: 233–238. 19066188 10.1136/jech.2008.078972

[13] Volmink J, Garner P (2007) Directly observed therapy for treating tuberculosis. Cochrane Database Syst Rev: CD003343. 10.1002/14651858.CD003343.pub3 17943789

[14] Grimwade K, Sturm AW, Nunn AJ, Mbatha D, Zungu D, et al (2005) Effectiveness of cotrimoxazole prophylaxis on mortality in adults with tuberculosis in rural South Africa. Aids 19: 163–168. 15668541 10.1097/00002030-200501280-00008

[15] Nunn AJ, Mwaba P, Chintu C, Mwinga A, Darbyshire JH, et al (2008) Role of co-trimoxazole prophylaxis in reducing mortality in HIV infected adults being treated for tuberculosis: randomised clinical trial. Bmj 337: a257. 18617486 10.1136/bmj.a257 PMC2656923

[16] Dheda K, Lampe FC, Johnson MA, Lipman MC (2004) Outcome of HIV-associated tuberculosis in the era of highly active antiretroviral therapy. J Infect Dis 190: 1670–1676. 15478074 10.1086/424676

[17] Tabarsi P, Saber-Tehrani AS, Baghaei P, Padyab M, Mansouri D, et al (2009) Early initiation of antiretroviral therapy results in decreased morbidity and mortality among patients with TB and HIV. J Int AIDS Soc 12: 14. 19607726 10.1186/1758-2652-12-14 PMC2734561

[18] Davis JL, Worodria W, Kisembo H, Metcalfe JZ, Cattamanchi A, et al (2010) Clinical and radiographic factors do not accurately diagnose smear-negative tuberculosis in HIV-infected inpatients in Uganda: a cross-sectional study. PLoS One 5: e9859. 20361038 10.1371/journal.pone.0009859 PMC2845634

[19] Diagnostic Standards and Classification of Tuberculosis in Adults and Children. This official statement of the American Thoracic Society and the Centers for Disease Control and Prevention was adopted by the ATS Board of Directors, July 1999. This statement was endorsed by the Council of the Infectious Disease Society of America, September 1999. Am J Respir Crit Care Med 161: 1376–1395. 10.1164/ajrccm.161.4.16141 10764337

[20] (2010) MANUAL OF THE NATIONAL TUBERCULOSIS AND. LEPROSY PROGRAMME. 2 nd. EDITION. 2010 Kampala.

[21] (2010) Stop TB initiative. Treatment of tuberculosis: guidelines 2010: World Health Organization. 23741786

[22] Erhabor GE, Adewole OO, Ogunlade O (2006) A five-year review of tuberculosis mortality amongst hospitalised patients in Ile-Ife. Indian J Chest Dis Allied Sci 48: 253–256. 16970290

[23] Hansel NN, Merriman B, Haponik EF, Diette GB (2004) Hospitalizations for tuberculosis in the United States in 2000: predictors of in-hospital mortality. Chest 126: 1079–1086. 15486367 10.1378/chest.126.4.1079

[24] Lubart E, Lidgi M, Leibovitz A, Rabinovitz C, Segal R (2007) Mortality of patients hospitalized for active tuberculosis in Israel. The Israel Medical Association journal: IMAJ 9: 870. 18210928

[25] (1996) Bactec 460 System: Product and Procedure Manual. Sparks, MD: Becton, Dickenson, and Company.

[26] (1999) Bactec MGIT 960 system user's manual. Sparks, MD: Becton, Dickenson, and Company.

[27] Muhumuza J, Asiimwe BB, Kayes S, Mugyenyi R, Whalen C, et al (2006) Introduction of an in-house PCR for routine identification of M. tuberculosis in a low-income country. Int J Tuberc Lung Dis 10: 1262–1267. 17131786

[28] World Health Organization. (2009) Treatment of tuberculosis: guidelines — 4th ed. Geneva: World Health Organization.

[29] Greenland S (2008) The need for reorientation toward cost-effective prediction: comments on 'Evaluating the added predictive ability of a new marker: From area under the ROC curve to reclassification and beyond' by M. J. Pencina et al., Statistics in Medicine (DOI: 10.1002/sim.2929). Stat Med 27: 199–206. 10.1002/sim.2995 17729377

[30] Grambsch PM, Therneau TM (1994) Proportional hazards tests and diagnostics based on weighted residuals. Biometrika 81: 515–526.

[31] Burton NT, Forson A, Lurie MN, Kudzawu S, Kwarteng E, et al (2011) Factors associated with mortality and default among patients with tuberculosis attending a teaching hospital clinic in Accra, Ghana. Trans R Soc Trop Med Hyg 105: 675–682. 21920570 10.1016/j.trstmh.2011.07.017

[32] van't Hoog AH, Williamson J, Sewe M, Mboya P, Odeny LO, et al (2012) Risk factors for excess mortality and death in adults with tuberculosis in Western Kenya. Int J Tuberc Lung Dis 16: 1649–1656. 23131264 10.5588/ijtld.12.0135

[33] Henegar C, Behets F, Vanden Driessche K, Tabala M, Bahati E, et al (2012) Mortality among tuberculosis patients in the Democratic Republic of Congo. Int J Tuberc Lung Dis 16: 1199–1204. 22871326 10.5588/ijtld.11.0613

[34] Silva DR, Menegotto DM, Schulz LF, Gazzana MB, Dalcin Pde T (2010) Factors associated with mortality in hospitalized patients with newly diagnosed tuberculosis. Lung 188: 33–41. 20131479 10.1007/s00408-009-9224-9

[35] Waitt CJ, Squire SB (2011) A systematic review of risk factors for death in adults during and after tuberculosis treatment. Int J Tuberc Lung Dis 15: 871–885. 21496360 10.5588/ijtld.10.0352

[36] Lopez-Gatell H, Cole SR, Hessol NA, French AL, Greenblatt RM, et al (2007) Effect of tuberculosis on the survival of women infected with human immunodeficiency virus. Am J Epidemiol 165: 1134–1142. 17339383 10.1093/aje/kwk116

[37] Rao VK, Iademarco EP, Fraser VJ, Kollef MH (1998) The impact of comorbidity on mortality following in-hospital diagnosis of tuberculosis. Chest 114: 1244–1252. 9823996 10.1378/chest.114.5.1244

[38] Taylor Z, Marks SM, Rios Burrows NM, Weis SE, Stricof RL, et al (2000) Causes and costs of hospitalization of tuberculosis patients in the United States. Int J Tuberc Lung Dis 4: 931–939. 11055760 PMC5448276

[39] Shimazaki T, Marte SD, Saludar NR, Dimaano EM, Salva EP, et al (2013) Risk factors for death among hospitalised tuberculosis patients in poor urban areas in Manila, The Philippines. Int J Tuberc Lung Dis 17: 1420–1426. 24125445 10.5588/ijtld.12.0848

[40] Greenaway C, Menzies D, Fanning A, Grewal R, Yuan L, et al (2002) Delay in diagnosis among hospitalized patients with active tuberculosis—predictors and outcomes. Am J Respir Crit Care Med 165: 927–933. 11934716 10.1164/ajrccm.165.7.2107040

[41] Pablos-Mendez A, Sterling TR, Frieden TR (1996) The relationship between delayed or incomplete treatment and all-cause mortality in patients with tuberculosis. JAMA 276: 1223–1228. 8849749 10.1001/jama.1996.03540150025026

[42] Abdool Karim SS, Naidoo K, Grobler A, Padayatchi N, Baxter C, et al (2011) Integration of antiretroviral therapy with tuberculosis treatment. N Engl J Med 365: 1492–1501. 22010915 10.1056/NEJMoa1014181 PMC3233684

[43] Blanc FX, Sok T, Laureillard D, Borand L, Rekacewicz C, et al (2011) Earlier versus later start of antiretroviral therapy in HIV-infected adults with tuberculosis. New England Journal of Medicine 365: 1471–1481. 22010913 10.1056/NEJMoa1013911 PMC4879711

[44] Havlir DV, Kendall MA, Ive P, Kumwenda J, Swindells S, et al (2011) Timing of antiretroviral therapy for HIV-1 infection and tuberculosis. New England Journal of Medicine 365: 1482–1491. 22010914 10.1056/NEJMoa1013607 PMC3327101

[45] Abdool Karim S, Naidoo K, Grobler A, Padayatchi N, Baxter C, et al (2010) Effect of initiating antiretroviral therapy during tuberculosis treatment in HIV-infected individuals: results of a randomized controlled trial in TB-HIV co-infected patients in South Africa (SAPiT Study). N Engl J Med 362: 697–706.20181971

